# Determinants of Israeli consumers’ decision to use food label information more frequently: a national survey study

**DOI:** 10.1186/s13584-021-00462-0

**Published:** 2021-03-15

**Authors:** Shosh Shahrabani

**Affiliations:** Department of Economics and Management, The Max Stern Yezreel Valley College, P.O. 1930600, Jezreel Valley, Israel

**Keywords:** Nutrition, Health belief model, Food labeling

## Abstract

**Background:**

Food labeling to encourage healthier food choices may have positive long-term effects. Yet previous studies point to challenges in terms of how consumers understand and use labeling information. The current study seeks to determine how psychological factors related to health and nutrition, food label perceptions, nutrition habits and sociodemographic factors are associated with consumers’ decisions to seek and use the information on nutrition food labels more frequently.

**Methods:**

The survey was conducted by a polling company in Israel between November 11 and December 12, 2019. Participants comprised a representative sample of the adult population in Israel age 21 and over. A total of 513 people returned the completed questionnaires, which included the following parts: personal details; frequency of searching for information on food product labels; perceived health risks of foods high in sodium, sugar and saturated fats; Health Belief Model constructs related to food labeling. The statistical analysis entailed ordinal logistics regressions.

**Results:**

While 59.3% of the sample reported that the information on food labels often or always affects their decision to purchase a food product, more than 20% reported often or always consuming products that are high in sugar (32.0%), saturated fats (31.3%) and salt (20.4%). The results of the analytical model show the following HBM variables to be significantly associated with frequency of using information on food labels: higher levels of perceived benefit (OR = 1.72, CI 95% = 1.12–2.64); higher confidence in reading and understanding food labels (OR = 2.48, CI 95% = 1.62–3.78); and higher perceptions of the importance of nutrition (OR = 2.76, CI 95% =1.97–3.87). In addition, women and married people were found to use food label information more frequently.

**Conclusions:**

General public information campaigns and HMOs campaigns with messages to motivate the use of food labels should emphasize the benefits of using labels to select food products. These messages should aim at increasing individuals’ perceived confidence in understanding the information on nutrition labels. The recent front-of-package labeling reform could be an important policy step for achieving healthier nutrition.

**Supplementary Information:**

The online version contains supplementary material available at 10.1186/s13584-021-00462-0.

## Background

In view of the major importance of nutrition in improving society’s health, most Western countries have invested abundant resources in national nutrition programs designed to motivate people to adopt healthier nutrition patterns [1, 2]. One of these programs entails mandatory food labeling [3, 4]. According to the National Academy of Medicine [5], nutrition labeling on food packages should provide information on energy (calories), saturated fat, trans fat, sodium and sugar. Food labeling is considered a tool [6, 7] and a cost-effective intervention for facilitating healthier food choices [8]. Product labeling may have positive long-term effects as it encourages companies to use healthier ingredients in their products, thereby creating a whole new supply of healthier food products [9, 10]. Yet previous studies point to challenges in terms of consumers’ ability to understand and properly use labeling information [11].

A review article found that the use of food labels varies significantly among different population groups [11]. People with lower socioeconomic status tend to read labels less often. This is especially problematic because low SES is linked to a higher risk of weight gain and obesity [12–14]. Moreover, research found that individuals whose diets are low in fat have a greater tendency to seek label information regarding the amount of fat in products than individuals whose diets are high in fat [15].

Data from Israel show that 50.9% of the Israel population aged 15+ are overweight or obese [16]. A 2014–2016 study conducted by the Israel Center for Disease Control (ICDC) to examine the health and nutrition status of Israelis between the ages of 25 and 64 [17] showed that the incidence of obesity is higher among lower socioeconomic classes. In turn, this obesity leads to an increase in morbidity rates from chronic diseases such as diabetes and cardiovascular diseases. The survey found that 81.9% of participants consumed sodium at levels higher than recommended and that the daily sodium consumption in Israel is approximately 112% higher for men and 53% higher for women than the values recommended by the US Dietary Reference Intake (DRI). Moreover, while the recommendation for adults is 50 g of added sugar per day, the average daily total sugar intake in Israel is 71 g for men and 60 g for women [17]. In addition, 41.3% of Israeli adults consume above the recommendation of the US Institute of Medicine, that saturated fat should compose no more than 7–10% of adults’ total daily calorie intake [17].

Since 1993, Israel has required all food manufacturers to label their products with nutrition information, yet only 42% of Israel’s population examines the ingredients or nutritional information provided on food packages [18]. Therefore, the Ministry of Health regulatory committee proposed legislation requiring manufacturers to place a red label on products high in saturated fats, sugar or sodium (negative) and a green label on category-specific best choices (positive) [19]. The new food labeling reform went into effect in Israel in January 2020.

The current study, conducted before the new labeling reform went into effect, provides new insights to the existing literature about the main factors associated with whether Israelis seek and use the information on food labels. Because Israeli society is culturally diverse, it is interesting to examine whether these factors differ from factors related to the use of food label information found in previous studies from other countries. In addition, the study uses the theoretical framework of the Health Belief Model (HBM), which very few previous studies implemented with respect to the use of food labeling in other countries. Understanding the factors that affect people’s use of food label information has important implications for nutrition education and public health.

## HBM and preventive health behavior

A large body of literature focuses on health beliefs and preventive health behavior based upon theoretical models, among them the Health Belief Model (HBM), reasoned action, social learning theory and others. These models provide information about people’s beliefs regarding health and health barriers. According to the social learning theory [20] of *response efficacy*, beliefs about whether a health behavior will lead to an expected outcome (e.g., the expectation that eating products high in sugar is related to health problems) are positively associated with the likelihood of performing the health behavior. According to the HBM [21] model, people are more motivated to carry out a particular health behavior when the perceived benefits of this behavior are higher (e.g., the perception that food label information helps them select healthier food). This is the case only if the perceived barriers for this behavior are low (e.g., reading labels does not take too much time) and the perceived confidence in carrying out this behavior is high (e.g., confidence in using the information on the label). Under such circumstances, people are more likely to utilize and act in accordance with the information on nutrition labels. The HBM model has been implemented in many studies in the field of health and also those examining the topic of food labeling [15, 22, 23].

In accordance with the HBM model, the findings of Lin et al. [15] for US data show that the probability of searching for information on food labels is positively correlated with the perceived benefits of using the information and negatively correlated with the perceived barriers to using the label information. In addition, individuals who feel strongly that what they eat can influence their risk of getting a disease (response efficacy) are more likely to use food label information [15, 22].

The purpose of the current study is to examine the factors associated with Israeli consumers’ decision to make use of food label information more frequently. In particular, the study uses empirical methods to examine the impact of the relationship between HBM principles and health behavior constructs on the frequency with which Israeli consumers search for and use food labeling information. Based on previous studies, the research hypotheses are:

*H1: HBM and preventive health behavior constructs:*

People will be more likely to use the information on food labels under the following conditions: (a) The *perceived benefits* of using food label information are higher. (b) The *perceived barriers* to using food label information are lower. (c) The level of *perceived confidence* in knowing how to use food label information in choosing a healthy diet increases. (d) The perceived *importance of nutrition* and choosing a healthy diet increases. (e) The level of *response efficacy*—i.e., awareness that consuming products high in fat/sugar/sodium is linked to health problems—increases. (f) The level of perceived risks of consuming unhealthy foods increases.

## Methods

The Institutional Review Board of the Max Stern Yezreel Valley College approved the current research. All participants gave their verbal informed consent, which was witnessed and formally recorded.

### Sample and sampling

Between November 11 and December 12, 2019, the B.I. and Lucille Cohen Institute for Public Opinion Research, a professional polling company, surveyed a representative sample of the adult population in Israel age 21 and over from the Jewish and non-Jewish sectors. The representativeness of the sample was examined based on distribution of the following socio-demographic characteristics compared with their distribution in the general population according to Central Bureau of Statistics (CBS) data: gender, age, level of education, level of religious observance, and religion. Constructing the sample entailed two stages: building a random sample of households according to the age criteria and locating telephone numbers for the sampled households. To ensure sample representativeness and high response rates, the polling company conducted the telephone survey 5 days a week, at different hours and on different days. Repeated trials were conducted on specific dates determined in accordance with the interviewees. In addition, the names of respondents who were not reached were recorded on a special list to control repeated trials (up to five repetitions per household) on different days and hours. The firm also made attempts to persuade those who initially refused to participate in the survey. Unfortunately, a telephone survey cannot provide demographic data for those who do not answer the survey. The gross sample included 1024 households that constituted the research population. The final sample included 513 respondents (50.1% response rate) who were interviewed by interviewers using the CATI system. Of the gross sample, 12 (1.2%) did not answer the phone, 472 (46.1%) refused to participate, and 25 (2.5%) answered partially and were dropped from the final sample.

### Questionnaire

The questionnaire included the following parts:

1. Personal details: socioeconomic information; age; marital status; education; nationality; year of immigration; degree of religious observance (1 = not at all religious, 5 = very religious); household income (1 = above average, 5 = much lower than average); number of persons in household; place of residence; employment status.
2. Perceived health status (1 = very good, 5 = very bad); chronic illness (Yes/No); weight (1 = much lower than normal weight, 5 = much higher than normal weight); extent to which participant maintains a normal body weight relative to age and height (1 = not at all, to 5 = to a very large extent); extent to which participant maintains a healthy diet low in sugar/salt/saturated fat (1 = not at all, 5 = to a large extent); frequency of food shopping (1 = every day, 5 = less than once every 2 weeks); responsible for home food purchasing (yes/no).
3. Frequency of searching for information on labels of purchased and consumed food products, on a scale of 1–5 (1 = not at all, 5 = always); frequency with which information on food labels affects participant’s decision to buy the product (1 = never, 5 = always);
4. Perceived health risk of food products high in sodium, sugar and saturated fat (1 = not at all, 5 = very high risk);
5. HBM constructs related to food labels, based on Lin et al. [15] and Gracia et al. [23] (see Table A1-Additional file 1). The scale ranged from 1 = strongly agree to 5 = strongly disagree. The constructs are: perceived benefits (e.g., food labels prevent fraud in food products); perceived barriers (e.g., food labels are not easy to understand); perceived importance of reading food labels (e.g., “using food labels to choose foods is better than relying on one’s own knowledge about what is in them”); confidence in reading the labels (“I am confident about knowing how to use food labels in choosing a healthy diet”); importance of nutrition (e.g., “It is important to take nutrition into account in shopping for food”); importance of diet (e.g., “It is important to choose a diet low in saturated fat”); response efficacy (“What you eat can make a big difference in your chances of getting a disease like heart disease or cancer”); health motivation (“I get periodic examinations every year, in addition to visiting the doctor when I am ill”).

The questionnaire was translated into Hebrew by the author and then back-translated by an English editor. In the first stage, a pilot questionnaire was administered to 50 individuals, and after improvements were made, the final format was developed.

### Statistical data analysis methods

SPSS 25 software was used for statistical analysis of the data. Fisher’s Exact test was used to define the relationship between the categorical variables and the dependent variable: frequency in using the information on food product labels in purchasing and consuming products. Results are considered significant if the two-sided *p*-value < 0.01, or *p* < 0.05, while they are considered marginally significant if *p* < 0.1. Cronbach alpha was calculated for the HBM constructs. Additionally, ordinal logistic regressions were used to identify the associations between the independent variables and the dependent variable.

## Results

Table A2 in the Additional file 1 shows the comparison between the distribution of the main demographic characteristics in the study sample and those in the Israeli adult population. The sample (participants age 21 and above) included 49.1% women and 50.9% men; 78.6% were Jews and 21.4% were Arabs and others; 46.4% were between the ages of 21–44, 34.3% between the ages of 45 and 64, and 19.3% age 65 and above. In comparison, the adult Israeli population over the age of 20 consists of 51.3% women and 48.7% men; 77.1% are Jews and 22.9% Arabs; 52.5% are between the ages of 20 and 44, 29.5% are between 45 and 64, and 17.9% are age 65 and above [24].

In the current study, 59.3% of the sample reported that the information on food labels often or always affects their decision to purchase a food product, while 22.8% said that the information never or rarely affects their decision. In addition, most of the participants reported that they maintain a balanced and healthy diet (69.9%). Yet more than 20% of the sample reported that they often or always consume products that are high in sugar (32.0%), saturated fats (31.3%) and salt (20.4%). Those who reported rarely or never eating a healthy diet included more men than women (64.7% versus 35.3%, respectively) and more people with 12 years of education or less than people with more than 12 years (54.9% versus 45.1%). Moreover, those who reported very often or always eating a healthy diet included more women than men (51.7% versus 48.3%, respectively) and more people with more than 12 years of education than people with less than 12 years of education (63.4% versus 36.6%). Table 1 summarizes the distribution of the demographic characteristics of the entire sample and shows the percentage of each characteristic according to frequency of using information on labels of purchased and consumed food products, coded as three categories: 1 = rarely/never, 2 = sometimes, and 3 = often/always.

**Table 1:** Survey data—frequency of using food labels according to socio-demographic and other characteristics (*N* = 513)

	Entire sample	Food labels affect buying decisions		
	%	Rarely/ Never (***N*** = 117)	Sometimes (***N*** = 92)	Often/ Always (***N*** = 304)
Gender				
Women	49.1%	18.3%	19.0%	62.7%*
Men	50.9%	27.2%	16.9%	55.9%
Age				
21–40	40.6%	24.6%	17.7%	57.6%
41–60	34.6%	21.4%	19.7%	59.0%
> 61	24.8%	22.6%	16.9%	60.5%
Religion				
Jewish	78.6%	23.8%	21.1%	55.1%***
Arab	21.4%	19.1%	6.4%	74.5%
Marital status				
Married	72.0%	19.1%	6.4%	74.5%*
Not married	28.0%	28.0%	20.3%	51.7%
Education				
Up to 12 years	39.4%	28.9%	18.4%	52.7%**
More than 12 years	60.6%	18.9%	17.6%	63.5%**
Residential region				
Tel Aviv and the center	48.3%	20.5%	23.4%	56.1%**
Other	51.7%	24.9%	13.4%	61.7%
Number of persons in household				
2 or less	36.3%	20.5%	20.0%	59.5%
3 and more	63.7%	24.4%	16.4%	59.3%
Maintaining a normal body weight^a^				
Not at all/ small/medium extent	35.7%	32.4%	18.1%	49.5%***
Large/very large extent	64.3%	17.7%	17.7%	64.6%
Responsible for shopping				
No	29.7%	27.6%	20.4%	52.0%*
Yes	70.3%	20.8%	16.9%	62.2%
Showing interest in healthy food intake				
Slightly/ Moderately	30.8%	45.9%	23.6%	30.6%***
Very much	69.2%	12.7%	15.3%	72.0%
Chronic illness				
No	77.8%	22.5%	17.4%	60.1%
Yes	22.2%	24.8%	20.4%	54.9%
Eat salty products				
Rarely/never/ sometimes	79.6%	19.9%	19.2%	60.9%***
Often/always	20.4%	34.6%	13.5%	51.9%
Frequency of eating sweets				
Rarely/never/ sometimes	68.0%	19.8%	17.2%	62.9%**
Often/always	32.0%	29.3%	19.5%	51.2%
Frequency of eating processed foods				
Rarely/never/ sometimes	90.6%	20.7%	18.1%	61.2%***
Often/always	9.4%	43.8%	16.7%	39.6%
Maintains a balanced and healthy diet				
Rarely/never/ sometimes	30.1%	46.1%	19.5%	34.4%***
Often/always	69.9%	12.9%	17.4%	69.7%

Two-sided *p*-value: ***p < 0.01, ** *p* < 0.05, * *p* < 0.1

^a^5-level scale ranging from (1) “not at all” to (5) “very much”

As can be seen in Table 1, the frequency of using the information on the labels of purchased and consumed food products is significantly higher among the following groups: those with higher education; Arabs compared to Jews; those who live in regions other than Tel Aviv and the center; those who usually maintain a normal body weight compared to those who rarely or never maintain a normal weight; those who follow a balanced and healthy diet; those who show interest in healthy food intake; and those who rarely or never eat salty or sweet products or processed foods. In addition, the findings in Table 1 indicate that the frequency of using the information on food product labels is marginally significant among women, among married compared to unmarried people, and among those who are responsible for shopping for food.

### Results for HBM categories and attitudes

Table 2 shows the distribution of the entire sample according to the HBM model categories and additional variables. The table also shows the percentage of each variable according to frequency of using the information on the labels of purchased and consumed food products. The Cronbach’s alpha coefficients are reported in Table A1 in the Additional file 1.

**Table 2:** The distribution of HBM variables, attitudes and other variables across categories of using food labeling (*N* = 513)

	Entire sample	Food labels affect buying decisions		
	%	Rarely/ Never (%) (***N*** = 117)	Sometimes (%) (***N*** = 92)	Often/ Always (%) (***N*** = 304)
Benefit 1: food labels prevent cheating				
Not agree at all/agree to small or medium extent	55.9%	26.8%	20.8%	52.4%***
Agree to a large /very large extent	441%.	15.6%	15.1%	69.3%
Benefit 2: food labels provide useful information				
Not agree at all/agree to small or medium extent	322%.	28.4%	23.5%	48.1%***
Agree to a large /very large extent	678%.	18.8%	15.5%	65.7%
Benefit 3: food labels ensure the quality and safety of food				
Not agree at all/agree to small or medium extent	575%.	25.9%	21.7%	52.4%***
Agree to a large /very large extent	425%.	16.1%	14.2%	69.7%
Barriers for using labels				
Not agree at all/agree to small or medium extent	64.1%	17.4%	17.1%	65.4%***
Agree to a large /very large extent	35.9%	31.1%	19.7%	49.2%
Perceived importance of reading food labels				
Not agree at all/agree to small or medium extent	49.2%	25.7%	18.3%	56.0%
Agree to a large /very large extent	50.8%	18.1%	18.5%	63.5%
Confidence of using labels				
Not agree at all/agree to small or medium extent	43.9%	31.5%	23.3%	45.2%***
Agree to a large /very large extent	56.1%	13.6%	14.3%	72.1%
Importance of healthy nutrition				
Not agree at all/agree to small or medium extent	4.3%	77.3%	9.1%	13.6%***
Agree to a large /very large extent	95.7%	20.4%	18.3%	61.3%
Response efficacy				
Not agree at all/agree to small or medium extent	16.7%	33.7%	22.9%	43.4%***
Agree to a large /very large extent	83.3%	19.1%	16.7%	64.3%
Health motivation				
Not agree at all/agree to small or medium extent	38.0%	28.9%	18.0%	53.1%**
Agree to a large /very large extent	62.0%	19.3%	18.0%	62.7%
Perceived risk level from unhealthy foods				
Very low/low /moderate risk	11.3%	42.1%	19.3%	38.6%***
High /very high risk	88.7%	20.3%	17.6%	62.1%

*** *p* < 0.01, ** *p* < 0.05, * *p* < 0.1

The results in Table 2 suggest that the frequency of using the information on food product labels is significantly higher for those who: a) perceive greater benefits of using food label information; b) perceive lower barriers to using food label information; c) have higher levels of confidence about knowing how to use food labels in choosing a healthy diet; d) perceive the importance of nutrition and diet to be greater; e) perceive response efficacy to be greater; f) perceive higher risks in consuming unhealthy foods; and g) have higher levels of health motivation.

### Analytical model results

Table 3 summarizes the results of the ordinal logistics regression analysis that examined the factors associated with the dependent variable: frequency of using the information on food product labels. Participants were asked the following question; “In general, how often does the information on food labels currently appearing on products influence your decisions whether to buy the product?” Participants’ responses were coded as three categories: 1 = rarely/never, 2 = sometimes, and 3 = often/always. The explanatory variables were: a) whether the participant is responsible for household food purchasing (yes/no); b) the HBM categories of perceived benefits, perceived importance of nutrition, and confidence in reading food labels (1 = do not agree and not sure, 2 = agree); c) the following socio-demographic variables: gender (base = men); age group (21–40 (base), 42–60, 61 and above); education level (12 years and less (base), more than 12 years); religion (Jews (base), non-Jews); marital status (married, not married (base)); and income (lower than average (base), average and above).

**Table 3:** Results of the regression analysis: factors associated with frequency of using food labels

Explanatory variables	Dependent variable: Food labels affect buying decision^b^					
	Model 1^**a**^			Model 2		
	OR	Confidence Interval 95%		OR	Confidence Interval 95%	
		Lower Bound	Upper Bound		Lower Bound	Upper Bound
Importance of healthy nutrition (base = Not at all/ Slightly/ Moderately)^c^	2.79***	2.04	3.84	2.76***	1.97	3.87
Confidence of using labels^b^ (base = Not at all/ Slightly/ Moderately)^c^	2.58***	1.76	3.77	2.48***	1.62	3.78
Responsible for shopping (base = No)^d^	1.55**	1.04	2.33	1.42	0.91	2.21
Benefit: food labels ensure the quality and safety of food (base = Do not agree/ In the middle)^e^	1.78***	1.20	2.64	1.72**	1.12	2.64
Gender (Base = Men)				1.58**	1.05	2.39
Age group 41–60 (Base = 21–40)				0.80	0.49	1.32
Age group above 60(Base = 21–40)				1.30	0.76	2.24
Education (Base = 12 years and less)				1.32	0.86	2.04
Religion (Base = Jews)				1.72*	0.98	3.01
Marital Status (Base = non-married)				1.66**	1.057	2.61
Income (Base = average and lower than average)				1.10	0.70	1.72
	**Pseudo R-Square** (Nagelkerke) = .204 Akaike’s Information Criterion (AIC) = 403.640			**Pseudo R-Square** (Nagelkerke) = .236 Akaike’s Information Criterion (AIC) = 723.127		

*** *p* < 0.01, ** *p* < 0.05, * *p* < 0.1

^a^Model 1 was adjusted for the variable “responsible for shopping”, while model 2 was adjusted for the variable “responsible for shopping”, and socio-demographic characteristics of gender, age group, education, religion, marital status and income

^b^The scale was 1 = Rarely/ Never, 2 = Sometimes, 3 = Almost always/ Always

^c^The scale was 1 = Not at all/ Slightly/ Moderately, 2 = Very much

^d^The scale was 0 = No, 1 = Yes

^e^The scale was 1 = Disagree/ In the middle, 2 = Agree

In several versions of the regression analyses, we used various HBM categories and control variables as a first step, among them socio-demographic variables and health status. We used the Akaike Information Criterion (AIC), which indicates the relative quality of statistical models for a given set of data. From among the candidate models, the models that minimized the AIC were chosen.

Table 3 includes two final models: Model 1, in which the independent variables were the HBM categories and whether the participant is responsible for household food purchasing, and Model 2, with the same independent variables as in Model 1, with the addition of the socio-demographic variables. The results of Model 1 in Table 3 show that after controlling for the rest of the explanatory variables, the following variables were significantly associated with the frequency of using the information on food product labels: a) the HBM categories of perceived benefit, confidence in reading food labels and importance of nutrition. More specifically, the chances of using the information on food product labels increase under the following conditions: the perceived benefit of food label use (“Food labels guarantee food quality and safety”) increases, the level of confidence in knowing how to use food labels in choosing a healthy diet increases, and the perceived level of importance of nutrition and diet increases. b) Those who are responsible for the household food purchasing are more likely to use the information on food product labels.

The results of Model 2 in Table 3 show that the following variables were significantly associated with the frequency of using the information on food product labels: the HBM category of perceived benefit, confidence in reading food labels and nutrition importance. In addition, the frequency of using food product label information was significantly higher among women than men and among those who are married than those who are not, and was marginally but significantly higher (*p* < 0.1) among non-Jews than Jews.

## Discussion

The current study examined a sample of Israeli adults to determine how psychological factors related to health and nutrition, food label perceptions, dietary intake, and sociodemographic factors are associated with self-reported use of food label information about nutrients. According to the findings, 59.3% reported that the information on food labels always or often affects their decision to purchase a particular food product, while 22.8% said that the information never or rarely affects their decision.

The univariate findings show that a higher proportion of those who reported using food labels more frequently have a higher level of education, live in regions other than Tel Aviv and the center, and are Arabs rather than Jews. In addition, according to the univariate findings, a higher proportion of those reporting using food label information more frequently maintain a normal body weight, follow a balanced and healthy diet, express interest in healthy food intake, are responsible for household food purchasing, and rarely or never eat salty, sweet or processed foods. The last result is compatible with the findings of Lin et al. [15] for the US, according to which individuals who consume more total fat, saturated fat or cholesterol are less likely to search for information on food labels than others. These findings can be explained by the cognitive dissonance model, according to which some people tend to ignore information that may cause cognitive dissonance between perceptions regarding a behavior and their own behavior [15].

Moreover, not only do individuals tend to disregard contradictory information, they also seek out congruent information to support their pre-existing food and/or food-related inclinations [25]. Therefore, even though food labels may provide important nutrition information that may help those with unhealthy nutrition habits, these people tend to ignore this information [15, 25]. These findings points to the need for policy steps to motivate people with unhealthy dietary habits to use food nutrition labels. One possibility is for HMOs to invite patients with chronic conditions (e.g., high blood sugar, high blood pressure) to participate in programs that encourage them to use food nutrition labels to purchase healthy food and improve their dietary habits.

The results of the analytical model that includes socio-demographic characteristics show that frequency of using the information on food product labels was significantly higher among women than men and among those who are married than those who are not, with the frequency among non-Jews higher by a marginal significance than among Jews. Yet, unlike previous studies conducted in other countries [12–14], we did not find any significant differences in the frequency of food label information use between those with low incomes and those with average and high incomes in Israel.

The results of the analytical model also confirm the validity of the HBM and other behavioral models with respect to the following constructs: perceived benefit of using food labels, perceived confidence in reading food labels, and perceived importance of nutrition. Those who made more frequent use of the information on food labels perceived the benefits of food labels to be greater, had higher levels of perceived confidence in knowing how to use food labels for choosing a healthy diet, and perceived the importance of nutrition and diet to be greater. These results are compatible with hypotheses H1(a), (c) and (d). In addition, these results for the Israeli population are in line with the findings of Rimpeekool et al. showing that the belief among Thai people that nutrition influences health increased their likelihood of using food label information to make decisions about food [22]. The results of the current study are also compatible with the findings of Jeong and Ham [26] for the South Korean population and of Lin et al. [15] for the US population, according to which perceived importance of healthy nutrition while buying food correlates with the probability of information-seeking. Lin et al. also found the following additional psychological factors to be connected to the probability of information-seeking: knowledge regarding nutrition and the correlation between excessive consumption of certain foods and health issues, and the perceived likelihood that a healthier diet actually decreases the chances of getting a chronic illness [15]. Nevertheless, contrary to Lin et al. [15], the current study did not find support for other HBM categories, such as perceived barriers, perceived importance of reading food labels and response efficacy. One possible explanation is that much has changed since the Lin et al. study was published in 2004, including greater access to online information that may improve people’s knowledge and attitudes with respect to nutrition.

The results of the current study imply that policy steps to increase individuals’ perceived confidence in understanding the information on nutrition labels may in turn increase their use of these labels. Moreover, messages to motivate the use of food labels should emphasize the benefits of using labels to select food products. In addition, these messages should aim at increasing individuals’ perceived confidence in understanding the information on nutrition labels and in turn at increasing their use of these labels.

A meta-analysis [27] that analyzed the findings of studies examining the effectiveness of different food labeling programs concluded that food labeling can have a significant impact on helping consumers make healthier food choices. Yet these studies also show that the ability to read and understand the printed information on food labels still constitutes a barrier in that it requires high levels of literacy and mathematical skills [28]. Therefore, food labeling systems must be easy to understand for all consumers [28]. Studies have found that consumers are able to more easily interpret and understand labeling methods that provide abbreviated information as compared to those that give detailed nutritional facts.

In fact, an important policy step that may be useful in achieving this goal is implementation of Israel’s recent front-of-package labeling reform. Front-of-package labeling uses two colors: red to signify a mandatory warning and green (voluntary) to signify food that complies with national nutrition recommendations [19, 29]. The new red/green labels are much easier for consumers to notice and read, hence potentially increasing consumers’ perceived confidence in understanding the information on nutrition labels and in turn encouraging them to purchase healthier products. This reform may help consumers make informed decisions at the point of purchase. Future studies can examine the impact of the new reform on food consumption and on public health in Israel.

The current study has some limitations. One is that the sample may have a selection bias since a telephone survey does not provide demographic data for those who did not answer the survey. The implication of this limitation concerns the representativeness of the sample, since non-respondents may have different characteristics than respondents. Yet, the response rate of 50.1% is relatively high compared to the 30% response rate typical of telephone surveys of this type [30]. In addition, the socio-demographic characteristics of the study sample are quite similar to those of the target Israeli adult population (Table A2 in the Additional file 1), indicating good representativeness of the sample. Another limitation of the study is that the survey included recall questions regarding individuals’ nutrition behavior. Recalled answers can be imprecise and affected by the social desirability bias. Future research is needed to follow up and examine the use of nutrition information on food labels after an intervention policy.

## Conclusions

The results of the current study indicate that health beliefs and behavioral factors are associated with the decision to use food label information frequently. In addition, socio-demographic factors and nutrition habits correlate with frequency of using information on food product labels. These findings may help policymakers design strategies for motivating individuals to use food nutrition labels and in turn to change their nutrition habits.

## Supplementary Information

**Additional file 1: Table A1.** HBM Constructs and Psychological Factors related to Food Labels. **Table A2.** Study Sample versus Israeli Population Distributions of Socio-demographic Characteristics.

## Data Availability

The dataset supporting the conclusions of this article is available from the authors upon request.

## Abbreviations

HBM: Health Belief Model
ICDC: Israel Center for Disease Control
CBS: Central Bureau of Statistics
