# Supplementary material for: Determinants of Israeli consumers’ decision to use food label information more frequently: a national survey study
Source: Isr J Health Policy Res. 2021 Mar 15;10:25. doi: 10.1186/s13584-021-00462-0 (PMC7971095; doi:10.1186/s13584-021-00462-0)
Supplement: Supplementary file 1 — Additional file 1: Table A1. HBM Constructs and Psychological Factors related to Food Labels. Table A2. Study Sample versus Israeli Population Distributions of Socio-demographic Characteristics. [file 13584_2021_462_MOESM1_ESM.docx]

**Additional file 1**

Table A1: HBM Constructs and Psychological Factors related to Food Labels

| **Categories** | **Items** |
| --- | --- |
| Perceived benefits | Food labels prevent fraud in food products |
|  | Food labels provide useful information |
|  | Food labels guarantee food quality and safety |
| Perceived Barriers | Nutrient information on food labels is hard to interpret |
|  | Food labels provide too much information |
|  | Food labels are often inconvenient to use |
|  | Reading food labels takes more time than one can spare |
| Perceived importance of reading food labels | Using food labels to choose foods is better than relying on one’s own knowledge about what is in them |
| Perceived Confidence | I am confident about knowing how to use food labels in choosing a healthy diet |
| Importance-nutrition and diet (alpha=0.716) | It is important to take nutrition into account in food shopping |
|  | It is important to adhere to a diet low in saturated fat |
|  | It is important to adhere to a diet low in sodium |
|  | It is important to adhere to a diet low in sugar |
|  | It is important to maintain a healthy weight |
| Response efficacy | What you eat can make a big difference in your chances of getting a disease like heart disease or cancer |
| Health motivation | I get periodic examinations every year, in addition to visiting the doctor when I am ill |

**Table A2: Study Sample versus Israeli Population Distributions of Socio-demographic Characteristics**

|  |  | CBS 2019 Census  Distribution in the population  Age 20 and above | The sample  Age 21 and above |
| --- | --- | --- | --- |
| Gender | Women | 51.3% | 49.1% |
|  | Men | 48.7% | 50.9% |
| Age group | 21-44 | 52.5%* | 46.4% |
|  | 45-64 | 29.5% | 34.3% |
|  | 65+ | 17.9% | 19.3% |
| Religion | Jews | 77.1% | 78.6% |
|  | Arabs and others | 22.9% | 21.4% |
| Education | 12 years and less | 40.3%** | 39.40% |
|  | More than 12 years | 59.7%** | 60.60% |
| Religious observance (Jews)** | Ultra-orthodox | 10.2% | 10.1% |
|  | Religious | 23.8% | 20.5% |
|  | Not religious | 65.7% | 68.4% |
|  | No answer | 0.3% | 1.0% |
| * In the population the age group is 20-44 while in the sample the age group is 21-44  ** Distribution in the population age 18 and above | | | |
